# Microbial Detoxification of Ochratoxin A in Food

**DOI:** 10.3390/foods15091551

**Published:** 2026-04-30

**Authors:** Elia Roncero, María J. Andrade, Micaela Álvarez

**Affiliations:** 1 Higiene y Seguridad Alimentaria, Instituto Universitario de Investigación de Carne y Productos Cárnicos, Facultad de Veterinaria, Universidad de Extremadura, 10003 Cáceres, Spain; eroncerob@unex.es; 2 Sección Departamental de Nutrición y Ciencia de los Alimentos, Facultad de Veterinaria, Universidad Complutense de Madrid, 28040 Madrid, Spain; malvar54@ucm.es

**Keywords:** adsorption, biodegradation, carboxypeptidase, ochratoxinase

## Abstract

Food contamination by ochratoxin A (OTA) constitutes a significant threat to public health and global food safety and security, a challenge increasingly intensified by climate change. Due to the high thermal and chemical stability of OTA, traditional physical and chemical decontamination methods often prove insufficient or detrimental to food quality. Consequently, microbial detoxification has emerged as a sustainable alternative. This review delves into the two primary biological mechanisms for OTA detoxification: physical adsorption—predominantly mediated by yeast and bacterial cell walls—and enzymatic biotransformation. Among the documented metabolic pathways, the hydrolysis of the amide bond by carboxypeptidases and amidohydrolases is recognised as the most reliable detoxification pathway. Conversely, alternative pathways, such as lactone ring opening, are hindered by their potential toxicity and chemical reversibility under acidic conditions. While various lactic acid bacteria, yeast, and filamentous mould species demonstrate high efficacy in OTA decontamination, their industrial implementation is currently limited by the complexity of food matrices and the lack of in vivo validation. The integration of multi-omics (proteomics and metabolomics), alongside CRISPR/Cas9 genome editing, is essential for identifying novel biocontrol agents. These precision biotechnological tools are fundamental for translating laboratory findings into industrial-scale OTA detoxification strategies.

## 1. Introduction

Consumption of food contaminated with mycotoxins poses a health risk for humans due to their potential toxic effects [1]. Consequently, many countries are applying legislation and guidelines to restrict the amounts of certain mycotoxins in food [2]. Furthermore, the presence of mycotoxin in food is expected to be exacerbated by climate change, which directly affects the spread of mould spores, insect infestations, and changes in the grains [1].

Once generated, mycotoxins are difficult to remove from foodstuffs due to their stability, and detoxification or mycotoxin removal is required. An efficient decontamination procedure implicates the elimination or neutralisation of mycotoxins while preventing the formation of toxic by-products, apart from maintaining organoleptic quality [1].

Thus, effective detoxification strategies include chemical and physical methods and microorganisms and their enzymes. Both advantages and drawbacks have been associated with all of them [2]. Some negative effects on the quality and safety of food have been potentially linked to chemical and physical tools. However, microbial detoxification is an environmentally friendly and low-cost method [1], with positive outcomes related to its effectiveness and impact on the quality of food matrices. In addition, the use of microorganisms for mycotoxin decontamination is especially valuable if they are already being used in food production [3].

Regarding microbial detoxification, bacteria, especially lactic acid bacteria, yeasts, and moulds, as well as isolated enzymes, have been reported for the biological reduction of mycotoxins, with greater decontamination abilities of bacteria than of fungi [1]. Apart from effectively decontaminating mycotoxins, the selected microorganism must meet several requirements, such as being safe to use, non-pathogenic, generating stable and well-characterised non-toxic metabolites, forming irreversible complexes, remaining active during the whole shelf life of the food, avoiding undesirable organoleptic features, preserving nutritional value, and requiring minimal resources for cultivation and production [4].

Microbial detoxification is exerted through adsorption and the breakdown of functional groups of the mycotoxins [1,5,6,7,8]. A variety of bacteria and yeasts have thus shown the ability to bind mycotoxins by the microbial wall [7,8,9,10]. Even though mechanisms involved in the microbial adsorption of mycotoxins are not well known, the structural integrity, physical structure and morphology, and chemical components of the microbial cell wall seem to be key factors influencing mycotoxin adsorption [11,12]. Additionally, microorganisms can transform mycotoxin into less toxic or non-toxic metabolites through reactions including glycosylation, hydrolysis, and deamination [7,12,13]. On the other hand, the study of these by-products is an important issue, as these can be equally or even more toxic than the original mycotoxin [14]. Among the more than 300 mycotoxins described, only data about the reduced toxicity of the metabolites derived from the microbial degradation of few mycotoxins, including ochratoxin A (OTA), aflatoxin B_1_ (AFB1), and deoxynivalenol (DON), compared with the parent mycotoxin have been reported in food [14,15,16]. Thus, the information available so far is scarce, and further research data about the toxicity of the degradation metabolites are required [1,6].

A limitation of using microorganisms for mycotoxin detoxification is that, in some cases, it can be a reversible process [8,9]. Moreover, the pathogenicity of the detoxifying microorganisms and the complexity of the food substrate must be assessed for industrial-scale implementation, and specific strategies should be formulated according to a given food substrate.

Considering that microbial detoxification of mycotoxins can be efficient, specific, and environmentally safer than the physical and chemical methods and given that it is less developed for OTA than for other mycotoxins, such as aflatoxins, this review aims to provide a comprehensive and critical overview of microbial detoxification mechanisms, key functional strains, enzymes, and prospects for industrial application in the control of OTA in foods. By synthesising the available knowledge, this work highlights the most effective biological strategies for mitigating OTA contamination in the food supply chain.

## 2. OTA Structure and Detoxification Mechanisms

### 2.1. OTA Structure

**Figure 1:**
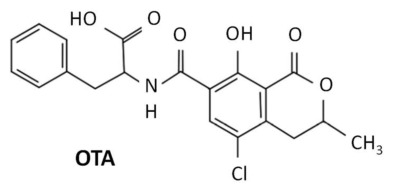
Chemical structure of ochratoxin A (OTA) adapted from Ben Miri et al. [18].

The chemical structure of OTA contains a molecule of L-phenylalanine linked to a dihydroisocoumarin moiety using an amide bond [17] (Figure 1, adapted from Ben Miri et al. [18]). The presence of a chlorine atom in OTA appears to be critical for its toxicity [19]. Regarding its physical properties, the melting point of OTA is around 168–173 °C. At lower temperatures, OTA is relatively stable, but reductions of more than 70% have been described at temperatures above 180 °C [19,20].

### 2.2. OTA Detoxification Mechanisms

Numerous studies have confirmed that bacteria, yeasts, and filamentous fungi can effectively detoxify OTA through degradation and/or adsorption [7,21].

#### 2.2.1. General Enzymatic Reactions

Enzymatic breakdown of mycotoxins occurs through a wide variety of chemical reactions. The literature summarises up to twelve main reaction types, including hydroxylation, oxidation–reduction, hydrogenation, de-epoxidation, methylation, glycosylation and glucuronidation, esterification, hydrolysis, sulphation, demethylation, and deamination [13].

Nevertheless, OTA is a challenging toxin because of its high chemical stability, which hinders its structural degradation. Thus, its enzymatic degradation primarily involves hydrolysis reactions catalysed by enzymes of the hydrolase class (EC 3) [5]. These reactions modify the substrate via nucleophilic catalysis, cleaving covalent bonds through the addition of water. Two primary hydrolytic pathways exist for OTA, with radically opposing toxicological implications [22]. Other secondary pathways include dechlorination (yielding ochratoxin B [OTB]) [23] and hydroxylation (forming hydroquinones) [24]. However, amide bond hydrolysis via carboxypeptidases and amidohydrolases remains the standard for biological detoxification.

##### A. Amide Bond Hydrolysis Mechanism

Amide bond hydrolysis represents the safest and most effective biodetoxification strategy documented to date [25,26]. It consists of the cleavage of the amide bond linking the isocoumarin group to the phenylalanine moiety. This reaction yields ochratoxin α (OTα) and L-β-phenylalanine [27]. OTα is regarded as virtually non-toxic (or significantly less toxic than the parental OTA) and is rapidly excreted, without accumulating in the kidney [28,29]. The enzymes responsible for this catalysis belong primarily to two families: carboxypeptidases (EC 3.4) and amidohydrolases (EC 3.5) [5,30].

*Carboxypeptidases (EC 3.4)*

Carboxypeptidases are exopeptidases that hydrolyse peptide bonds located at the C-terminal of proteins and peptides [31,32]. Their ability to degrade OTA is due to the toxin’s structure mimicking a dipeptide with a terminal phenylalanine residue. Carboxypeptidase A (CPA) was the first enzyme identified with the ability to detoxify OTA, described in the pioneering study by Pitout [33] using bovine pancreatic CPA. This metalloenzyme exhibits a preference for substrates with aromatic amino acids at the C-terminal. Conversely, carboxypeptidase Y (CPY), subsequently identified in *Saccharomyces cerevisiae* [30], unlike CPA, CPY is a vacuolar exopeptidase with optimal activity at an acidic pH (pH 5.6), although its reaction rate is slow due to its low specificity. Recent investigations have isolated homologous carboxypeptidase genes, specifically the *dacA* gene in bacteria such as *Bacillus subtilis* and *Acinetobacter* sp. [34].

*Amidohydrolases and amidases (EC 3.5)*

Enzymes that have demonstrated significantly higher catalytic efficiency than that of classical carboxypeptidases are included in this group [35]. These enzymes are often zinc-dependent metalloenzymes that act specifically on non-peptide or modified amide bonds, as is the case with the ochratoxinase from *Aspergillus niger* [32]. Dobritzsch et al. [36] purified the enzyme responsible for OTA degradation, demonstrating that it was not a lipase but a specific amidase (designated as AnOTA or ochratoxinase). This enzyme is a metal-dependent amidohydrolase that lacks general aminoacylase or endopeptidase activity, conferring high specificity for OTA. This explains why OTA (which contains phenylalanine) is an ideal substrate, whereas other peptides are not hydrolysed, affording these enzymes high selectivity in complex matrices such as foods.

Recently, Peng et al. [37] isolated four novel enzymes, BnOTases 1–4, from the *Brevundimonas naejangsanensis* ML17 strain. These enzymes are notable not only for degrading OTA but also for their capacity to hydrolyse ochratoxin B (OTB) into ochratoxin β (OTβ), thereby broadening the detoxification spectrum. The study of the newly purified enzymes provides key insights, revealing that BnOTase2 exhibits an extremely high affinity for OTA. This characteristic renders it a good candidate for removing traces of OTA at very low concentrations.

##### B. Lactone Ring Hydrolysis Mechanism

The opening of the lactone ring in the OTA molecule is a key mechanism for the detoxification of this mycotoxin, which involves the hydrolysis of the cyclic ester bond (lactone) within the isocoumarin structure [38]. The reaction is catalysed by enzymes known as lactonases or lactonohydrolases, which are common in various microorganisms [39]. The result is lactone-opened OTA (OP-OTA), chemically described as *N*-[3-carboxy-5-chloro-2-hydroxy-4-(2-hydroxypropyl)benzoyl]-L-phenylalanine and previously detected as a biodegradation product [40].

Unlike OTα, OP-OTA exhibits similar toxicity to the original OTA when administered to rats, although the toxicity is lower in mice [41]. Furthermore, there is a risk of reaction reversibility under acidic pH conditions, leading to ring re-closure and the regeneration of the active toxin [42].

Recent studies using LC/MS analysis have detected OP-OTα as a metabolite in the degradation carried out by strains such as *Bacillus velezensis* E2 and *B. amyloliquefaciens* YL-1 [43,44], suggesting it may be a subsequent step to amide bond cleavage.

Unlike the amide bond cleavage pathway (which generates non-toxic OTα), OP-OTA poses two major drawbacks in terms of food safety. Toxicological assays indicate that OP-OTA retains toxicity similar to that of the parent molecule (OTA) when administered to rats, although it appears to be less toxic in mouse models [41,42,45]. Therefore, this pathway does not ensure effective detoxification.

##### C. Other Enzymes

In addition to specific enzymes (ochratoxinases) and classical carboxypeptidases, the literature describes the capacity of other enzymatic families to degrade OTA. This is often attributed to enzymatic promiscuity, whereby enzymes targeting different substrates act on OTA with lower efficiency.

*Lipases (EC 3.1.1.3)*

Although the primary function of lipases involves hydrolysing ester bonds in lipids, certain commercial lipases have demonstrated activity towards the amide bond of OTA [46,47]. Concretely, *A. niger* lipase and porcine pancreatic lipase (PPL) have shown the ability to hydrolyse OTA and OTB [48]. Nonetheless, in-depth studies suggest that the activity observed in crude commercial lipase preparations is often attributable to the presence of amidase impurities rather than to the lipase *per se* [36].

*Serine proteases and cysteine proteases*

Based on in silico studies (molecular docking) and in vitro assays, trypsin and bromelain theoretically prove their ability to interact with OTA but exhibit low actual efficiency under acidic conditions. Docking studies indicate a lower affinity compared with specific metalloenzymes [49].

*Neutral metalloendopeptidases*

Among non-specific proteases, neutral metalloendopeptidases (particularly those from *B. subtilis*) have shown promising results as effective agents for breaking down this mycotoxin, with OTα as the end-product [49].

##### D. Hydroxylation

This process is catalysed primarily by enzymes of the Cytochrome P450 (CYP450) system in animals and certain fungi [13]. Hydroxylation typically occurs at the C-4 or C-10 positions, yielding 4*R*-hydroxyochratoxin A.

#### 2.2.2. Dechlorination

This reaction involves the elimination of the chlorine atom at the C-5 position of the isocoumarin ring. This yields OTB, which, although less toxic than OTA, is not innocuous. This process has been observed in renal microsomes from phenobarbital-treated rabbits and in certain bacteria [23].

#### 2.2.3. Peroxidation

Peroxidases (POs) derived from rice bran, which utilise hydrogen peroxide to oxidise OTA, have demonstrated potential for detoxifying the mycotoxin. However, it seems that this capacity is limited by environmental conditions. Thus, a modest reduction (17%) in mycotoxin levels was found in white grape juice when peroxidases were used, whereas no significant reduction was observed in red grape juice [50]. This suggests that factors such as pH and the presence of antioxidants limit the applicability of this oxidative pathway compared with the hydrolytic pathway.

#### 2.2.4. Adsorption

The cell wall of yeasts, mainly composed of -D-glucans, glucomannans, and mannan oligosaccharides, has shown effective removal of OTA from food by physical adsorption, even using inactivated cells [11,51]. It has been demonstrated that OTA removal is strain dependent and depends on the thickness and diameter of the cell wall [52]. Thus, the greater the cell wall content, the greater the ability to remove the mycotoxin [52]. Furthermore, one of the advantages of using yeast cell walls is that they do not significantly alter critical sensory and technological parameters when added to brewing wort and apple juice [51,53].

However, the adsorption efficiency depends not only on intrinsic factors but also on environmental conditions. Thus, differences in that parameter have been influenced by the dose employed, temperature, pH, and specific interactions with the matrices [51,52]. Additionally, the mycotoxin–yeast union has to remain stable after passing through the gastrointestinal tract since conditions there could enhance or break the bond, which would affect the bioavailability of OTA at the gut level [52].

On the other hand, bacterial biomass has also been described as an OTA adsorbent but to a lesser extent, maybe due to unstable binding [8]. In this context, the hydrophobic properties and electron donor–acceptors seem to be implicated in the higher binding of inactivated bacterial cells than living cells [8].

## 3. Microbial Detoxification Agents

In the last year, there has been an increase in published studies on OTA detoxification by microorganisms [51,54,55,56,57,58].

### 3.1. Bacteria

Traditionally, studies on the microbial detoxification of OTA have focused on the use of lactic acid bacteria, such as species belonging to the former genus *Lactobacillus* [8,59]. For example, Escrivá et al. [60] demonstrated the OTA reduction ability of different strains of *Lactiplantibacillus plantarum*, *Lactobacillus rhamnosus*, and *Lactobacillus paracasei* isolated from goat milk whey in liquid Man–Rogosa–Sharpe (Table 1). On the other hand, the combination of fermentation and thermal-pressure treatment in grape pomace resulted in up to 97% OTA reduction for *L. paracasei*, *Lactobacillus acidophilus*, and *Lp. plantarum* strains [54].

Another bacterium, *Brevundimonas diminuta* HAU429, isolated from vineyards, eliminated the mycotoxin by more than 40% using multiple hydrolases to generate OTα [61] (Table 1). As a drawback, this effect was dependent on pH and temperature.

Another example of an OTA-degrading enzyme isolated from bacteria is ADH3 from *Stenotrophomonas acidaminiphila*. An amount of 1.2 mg/mL of ADH3 completely degrades 50 mg/L OTA within 90 s [62] (Table 1). Due to the high potential for degrading OTA of this enzyme, Dai et al. [65] obtained the upgraded variant S88E expressed by *Pichia pastoris*, which exhibits 3.7-fold higher enzymatic activity. Two other OTA hydrolases structurally similar to ADH3, ADH1 and AMD3, from different bacterial strains (*Silanimonas* sp. CW282 and *Luteimonas* sp. CW574), have been identified and characterised and show high tolerance to NaCl environments [44,57]. The amidohydrolase *Bl*OTA from *Brevibacterium linens* DSM 20425^T^ detoxified the OTA from bovine milk and presented optimal activity at pH 8 [63]. The BnOTase1-4 from *B. naejangsanesis* isolated from soil were able to degrade 90% of OTA at 25–55 °C [66]. However, this bacterium was also able to reduce the OTA amounts not only by producing these enzymes but also through other unknown mechanisms [66].

Another example is *Acinetobacter* sp., which was able to degrade more than 70% of OTA (Table 1), an activity that was confirmed by expressing the involved carboxypeptidase in *Escherichia coli* [64]. The bacterium *B. subtilis* also produced a carboxypeptidase able to degrade 71.3% of the OTA amounts [34].

Furthermore, the detoxification ability of *Lactobacillus* has been tested after the cells died. For example, the dead cells of *Lp. plantarum*, *Levilactobacillus brevis*, and *Fructilactobacillus sanfranciscensis* were able to detoxify higher levels of OTA than live cells, up to 58.82 ± 0.56 [8].

In summary, research in recent years demonstrates a significant shift from the use of whole-bacterial biomass toward the identification and characterisation of high-efficiency specific enzymes, such as amidohydrolases and carboxypeptidases. While adsorption by lactic acid bacteria remains a valuable tool due to their “Generally Recognised as safe” (GRAS) status, its effectiveness is often limited by reversibility and environmental sensitivity. In contrast, the emergence of isolated enzymes like ADH3 or *Bn*OTase offers a path toward the complete and irreversible degradation of OTA.

### 3.2. Yeasts

The variety of yeasts found to have the ability to detoxify OTA has been lower than that of bacteria, and, in most studies, this has been related to adsorption (Table 2). The detoxifying capacity of *S. cerevisiae* has thus been well established for years, being mainly attributed to its cell wall adsorption mechanisms [67]. Consequently, some commercial bioadsorbents of mycotoxins, including yeast cell wall components, are available. For example, Mycosorb^®^ includes cell wall elements from *S. cerevisiae* isolated from beer fermentation that have demonstrated an overall mean OTA adsorption efficiency of around 32.28% [68].

Recently, the residual biomass of *S. cerevisiae* from industrial beer fermentation has also been proposed as a bioadsorbent [51]. As a disadvantage, the maximum OTA removal was only 14% using 20 mg of bioadsorbent [51].

Other authors suggest that *S. cerevisiae* can produce CPY, which is able to transform 52% of the OTA into OTα [46].

The adsorption has also been described as the main mechanism of OTA detoxification by other yeasts, such as *Debaryomyces hansenii* and *Rhodosporidiobolus ruineniae* [69,71]. For example, *R. ruineniae* reduced the amount of OTA by 50% after 1 h of incubation, although the binding to the cell wall was weak [71]. In addition, pH 3 has been described as favouring the ionisation of both cell wall molecules from *D. hansenii* and OTA, strengthening their binding affinity and, consequently, enhancing the detoxification of the medium [69]. On the contrary, neutral pH has shown better detoxification levels by *Hanseniaspora uvarum* than that at pH 3, although pH 5.5 presented similar detoxification percentages [70]. These findings suggest that, while adsorption is a conserved mechanism for OTA detoxification across various yeast species, the optimal pH for binding is highly species dependent.

Regarding other yeast species, *Meyerozyma caribbica*, for example, seems to employ different modes of action than the previous yeasts, since it can reduce OTA concentrations due to degradation pathways that produce metabolites other than OTα [71].

Other examples of yeasts capable of detoxifying OTA are the strains of *Kazachstania unisporus* AC-2 and YL-8 isolated from kefir, which were able to remove approximately 46.1 and 40.2% of OTA in yeast extract peptone dextrose (YPD) medium, respectively [72]. However, the mechanisms of action of these strains were not analysed. One advantage of these strains was their ability to tolerate acid and bile salt environments.

Finally, *Lachancea thermotolerans* has shown its ability to detoxify OTA during wine fermentation, although the effect is dependent on the strain used [55]. The detoxification ranges varied from 54.01 ± 4.07% to 91.36 ± 0.32% [55].

Overall, the recent literature confirms that yeast-based detoxification continues to rely predominantly on physical adsorption within the cell wall, with *Saccharomyces cerevisiae* remaining the industrial benchmark.

### 3.3. Moulds

The ability of OTA detoxification has also been attributed to filamentous moulds, with their activity being associated with enzymatic degradation (Table 3).

Two species of *Metarhizium* presented an OTA-like cluster and the total ability to degrade OTA and the majority of the OTB [58]. The responsible mechanism was the production of amidohydrolases MbAmh1 in *Metarhizium brunneum* and its homolog in *Metarhizium robertsii*, namely MrAmh1. However, as described for other enzymes, the effect was dependent on pH, being unable to degrade OTA at pH 3 and 10 [58]. These proteins are structurally similar to the most famous amidohydrolase, ochratoxinase from *A. niger* [58], which hydrolyses OTA, OTB, and substrates containing phenylalanine, alanine, and leucine residues at their C-terminal position [32]. *A. niger* lipases also showed the ability to degrade OTA with high specificity in 3 h [48]. Moreover, OTA was fully hydrolysed by the ochratoxinase *An*OTA from *A. niger* when it was added to three plant-based beverages (almond, soy, and oat) incubated for 4 h at 20 and 37 °C [32].

In conclusion, unlike bacteria and yeasts, the OTA detoxification potential of filamentous moulds is almost exclusively driven by enzymatic hydrolysis. The discovery of specific gene clusters and specialised enzymes such as A*n*OTA, highlights an evolutionary specialisation toward the irreversible conversion of OTA into non-toxic metabolites. However, the direct application of moulds in food processing is severely restricted by the risk of secondary mycotoxin production. Consequently, future research is increasingly pivoting toward the use of these fungal enzymes rather than the original fungal strains.

## 4. Future Perspectives

The microbial detoxification of OTA represents a promising frontier for food safety; however, several critical gaps must be addressed to transition from laboratory-scale findings to industrial applications.

Future research must prioritise the “real-world” challenge posed by complex food matrices, given that the most positive outcomes have been recorded in controlled environments. Future research must thus focus on foodstuffs in which proteins, fats, and polyphenols can interfere with enzyme stability or binding sites [48]. Moreover, enzymatic stability under industrial processing conditions—such as pasteurisation—remains a critical factor that must be optimised before commercial adoption. Furthermore, in vivo studies are crucial to ensure that detoxification remains effective under the fluctuating pH and enzymatic conditions of human and animal gastrointestinal tracts [56,68]. Therefore, it is necessary to combine gut simulation with cellular assays to monitor the stability of the mycotoxin–adsorbent complex and ensure detoxification rates [68]. The transition to an industrial scale also requires careful cost–benefit analysis, as it is necessary to address the high production costs associated with enzyme purification. In this regard, the use of microbial extracts or fermentation by-products suitable for human consumption could offer a more cost-effective strategy for large-scale applications. In addition, the potential impact of microbial treatments on the organoleptic properties of food must be minimised. Therefore, developing low-cost cultivation strategies and using GRAS microorganisms is essential for the commercial adoption of these biotechnological solutions.

While several enzymes like ochratoxinases and carboxypeptidases have been well characterised, many microorganisms show significant OTA reduction through mechanisms that remain unknown. Identifying these specific metabolites and the metabolic pathways involved could lead to the discovery of more-robust and efficient bio-detoxifying agents. In this context, the use of metabolomics and proteomics shifts the focus from observing to understanding OTA degradation by identifying key pathways and enzymes [61,73]. These techniques, combined with CRISPR/Cas9, enable the precise engineering of food-grade strains to overexpress detoxification genes and optimise enzyme stability for industrial applications [74].

Finally, the degradation of OTA into other metabolites can produce metabolites with residual toxicity [75] or the potential to revert to the parent toxin [8]. Future research must focus on high-throughput toxicological assays to guarantee that any biocontrol treatment results in a truly safe end-product.

## 5. Conclusions

Microbial detoxification represents a highly promising alternative to traditional physical and chemical methods for mitigating OTA, characterised by high specificity, sustainability, and the preservation of the sensory quality of foods.

Within the mechanisms used by microorganism to detoxify OTA, the adsorption one provides an immediate reduction in OTA levels, although its effectiveness is often hindered by its reversible nature. Consequently, enzymatic hydrolysis of the amide bond seems to be the most effective mechanism for OTA detoxification.

To achieve industrial-scale implementation of microbial detoxification, future research must bridge the gap between laboratory media and complex food matrices, ensuring the stability and safety of the process under in vivo conditions and during food manufacturing. The integration of multi-omics and CRISPR/Cas9 will be decisive in engineering, food-grade strains and high-efficiency enzymes, providing a precise biotechnological response to the global health challenge posed by mycotoxins in a changing climate.

## Figures and Tables

**Table 1:** Bacteria able to detoxify ochratoxin A, modes of action, and substrate.

Bacteria	Mode of Action	Substrate	Detoxification Rate	Reference
*Lactiplantibacillus plantarum*, *Levilactobacillus brevis*, and *Fructilactobacillus sanfranciscensis*	Adsorption	Phosphate buffered saline (PBS)	Dead cells: 46.2 to 59.8%	[8]
*Lactobacillus bulgaricus LB6*, *Lacticaseibacillus paracasei BAA52*, *Lactobacillus* *acidophilus*, and *Lp. plantarum*	Enzymatic degradation and adsorption	Grape pomace	*L. bulgaricus*: 6.7 to 13.82% *L. paracasei*, *L. acidophilus*, and *Lp. plantarum*: 62–66%	[54]
*Brevundimonas diminuta*	Hydrolases	PBS	Cell lysate: 100% Free cell supernatant: 53%	[61]
*Lp. plantarum*, *Lactobacillus* *rhamnosus*, and *Lactobacillus* *paracasei*	-	Man–Rogosa–Sharpe	9.4–39.9%	[60]
*Stenotrophomonas acidaminiphila*	Amidohydrolase ADH3	PBS	100%	[62]
*Silanimonas* spp. CW282 and *Luteimonas* spp. CW574	Amidohydrolases ADH1 and AMD3 expressed by *Pichia pastoris*	Methanol	ADH1: 5 to 13% ADM3: 35 to 63%	[44]
*Luteimonas* spp. CW574	Amidohydrolase AMD3	Nutrient broth	50% at 72 h	[57]
*Brevibacterium linens* DSM 20425T	*Bl*OTA	PBS and milk	100%	[63]
*Brevundimonas naejangsanesis*	*Bn*OTase1-4	-	100%	[37]
*Acinetobacter* sp. *neg1*	Carboxypeptidase PJ_1540	Minimal medium peptone	>70% at 144 h	[64]
*Bacillus subtilis*	Carboxypeptidase	Ethylenediaminetetraacetic acid (EDTA) with metal ions	71.3% at 37 °C for 24 h	[34]

-: Absence of information.

**Table 2:** Yeasts able to detoxify ochratoxin A, modes of action, and substrate.

Yeast	Mode of Action	Substrate	Detoxification Rate	Reference
*Saccharomyces* *cerevisiae*	Adsorption	Brewery wort	Up to 14.11%	[51]
*Debaryomyces hansenii*	Adsorption	Phosphate buffered saline (PBS)	Up to 100% at pH 3	[69]
*S. cerevisiae*	Adsorption	Buffer solution	32.28 ± 7.11%	[68]
*Hanseniaspora uvarum*	Adsorption and other active mechanisms	Potato dextrose broth	Up to 82.96 ± 1.14%	[70]
*Rhodosporidiobolus ruineniae* and *Meyerozyma caribbica*	*R. ruineiae*: adsorption *M. caribbica*: degradation	Potato dextrose broth	*R. ruineniae*: >50% at 1 h *M. caribbica*: >33% at 24 h	[71]
*Kazachstania* *unisporus*	-	Yeast extract peptone dextrose	41.6–40.2% at 24 h	[72]
*Lachancea* *thermotolerans* and *S. cerevisiae*	-	Wine	*Lachancea* *thermotolerans*: 54.01 ± 4.07% to 91.36 ± 0.32% *S. cerevisiae*: 75.85 ± 0.89%	[55]

-: Absence of information.

**Table 3:** Moulds’ ability to detoxify OTA, modes of action, and substrate.

Moulds	Mode of Action	Substrate	Detoxification Rate	Reference
*Metarhizium brunneum* and *Metarhizium robertsii*	Amidohydrolases MbAmh1 and MrAmh1	Reaction system with Tris-HCl buffer	100% at 12 h	[58]
*Aspergillus niger*	Ochratoxinase *An*OTA	Almond, oat, and soy beverages	100%	[32]
*A. niger*	Lipase ANL	Phosphate buffer	100%	[48]

## Data Availability

No new data were created or analysed in this study. Data sharing is not applicable to this article.
